# Association between hOGG1 polymorphism rs1052133 and gastric cancer

**DOI:** 10.18632/oncotarget.16124

**Published:** 2017-03-11

**Authors:** Dingding Zhang, Xiaoxin Guo, Jinliang Hu, Guangqun Zeng, Maomin Huang, Dandan Qi, Bo Gong

**Affiliations:** ^1^ Sichuan Provincial Key Laboratory for Disease Gene Study, Hospital of University of Electronic Science and Technology of China and Sichuan Provincial People's Hospital, University of Electronic Science and Technology of China, Chengdu, Sichuan, 610072, China; ^2^ Institute of Health Policy and Hospital Management, Sichuan Academy of Medical Science and Sichuan Provincial People's Hospital, Chengdu, Sichuan, 610072, China; ^3^ School of Public Health, Sichuan University, Chengdu, Sichuan, 610041, China; ^4^ Department of Clinical Laboratory, People's Hospital of Pengzhou, Pengzhou, Sichuan, 611930, China; ^5^ Department of Immunology, Zunyi Medical College, Zunyi, Guizhou, 563000, China

**Keywords:** gastric cancer, meta-analysis, hOGG1 rs1052133, association

## Abstract

**Purpose:**

To conduct a comprehensive evaluation of the association of the *human8-oxoguanine glycosylase 1* (*hOGG1*) gene polymorphism rs1052133 with gastric cancer (GC) through a systematic review and meta-analysis of genetic association study.

**Results:**

A total of 15 articles from published papers were included in our analysis. The meta-analyses for *hOGG1* rs1052133, composed of 4024GC patients and 6022controls, showed low heterogeneity for the included populations in all the genetic models, except for the Caucasian population under allelic genetic model, the Asian population under addictive model and Caucasian population under dominant model. The analyses of all the genetic models in overall pooled populations did not identify any significant association between GC and *hOGG1* rs1052133 (Allelic model: C vs. G, *p* = 0.746; Addictive model: CC vs. GG, *p* = 0.888; Recessive model: CC +GC vs. GG, *p* = 0.628; Dominant model: CC vs. GG+GC, *p* = 0.147), even though stratified analyses were conducted in different ethnicities under each genetic model.

**Materials and Methods:**

All case-control association studies on *hOGG1* and GC reported up to December 15, 2016 in PubMed, Embase, Web of Science, and the Chinese Biomedical Database were retrieved. Odds ratios (ORs) and 95% confidence intervals (95% CIs) were calculated for single-nucleotide polymorphism (SNP) using fixed- and random- effects models according to between-study heterogeneity. Publication bias analyses were conducted using Begg test.

**Conclusions:**

This meta-analysis showed there was no association between *hOGG1* rs1052133 and GC. Given the limited sample size, further investigations including more ethnic groups are required to validate the association.

## INTRODUCTION

Gastric cancer (GC) is one of the most common cancers and the leading causes of cancer death in the world [1], however, there is a gradually decrease in incidence and mortality rates in most developed countries [2]. GC is a multifactorial disease caused by dietary, genetic and environmental factors [3, 4]. Although the pathogenetic mechanism of GC is very complicated and is still not fully understood, more and more evidence has been shown that there is a correlation between genetic polymorphisms and GC risk [5–7].

To date, many genetic polymorphisms in the carcinogen detoxification, antioxidant protection, DNA repair and cell proliferation processes have been reported to play a crucial role in the development of GC [8]. The *human 8-oxoguanine glycosylase 1* (*hOGG1*) gene, specifically involved in the repair of DNA oxidative damage in the base excision response pathway, has been shown to be associated with a variety of cancers [9–13]. As an important component of DNA repair pathway, *hOGG1* encodes a DNA glycosylase enzyme that actively removes 8-hydroy-2-deoxyguanine, which is highly mutagenic and a major form of oxidative DNA damage [14, 15]. The dysfunction of hOGG1 might result in the DNA repair deficiency and then induce gene mutation and cell canceration. Functional studies showed that the hOGG1 variant had normal enzymatic activity, but maintained greater sensitivity to oxidation than wildtype hOGG1 protein [16].

The *hOGG1* gene has been regarded as a candidate for involvement in the underlying cause of GC and the *hOGG1* polymorphism rs1052133 has been widely evaluated in association with GC across different ethnicities [11, 17–31]. *hOGG1* polymorphism rs1052133 has been reported to be associated with an altered risk for GC in Chinese, Japanese and Caucasian populations [11, 18, 19, 32]. However, other reported studies on the association between *hOGG1* rs1052133 and GC risk are inconclusive and conflicting [20–31]. Such inconsistence and heterogeneity could be caused by different sample sizes and diversities in multiple ethnic cohorts. Meta-analysis, which combined all studies with the same criteria, could be helpful to comprehensively explain the association of *hOGG1* rs1052133 with GC and provide some new clues for the research on GC. Therefore, in this study we conduct a systematic review and meta-analysis of all association studies on *hOGG1* rs1052133 with GC, to summarize and evaluate the association between *hOGG1* rs1052133 and GC.

## RESULTS

### Literature search and characteristics

**Figure 1:**
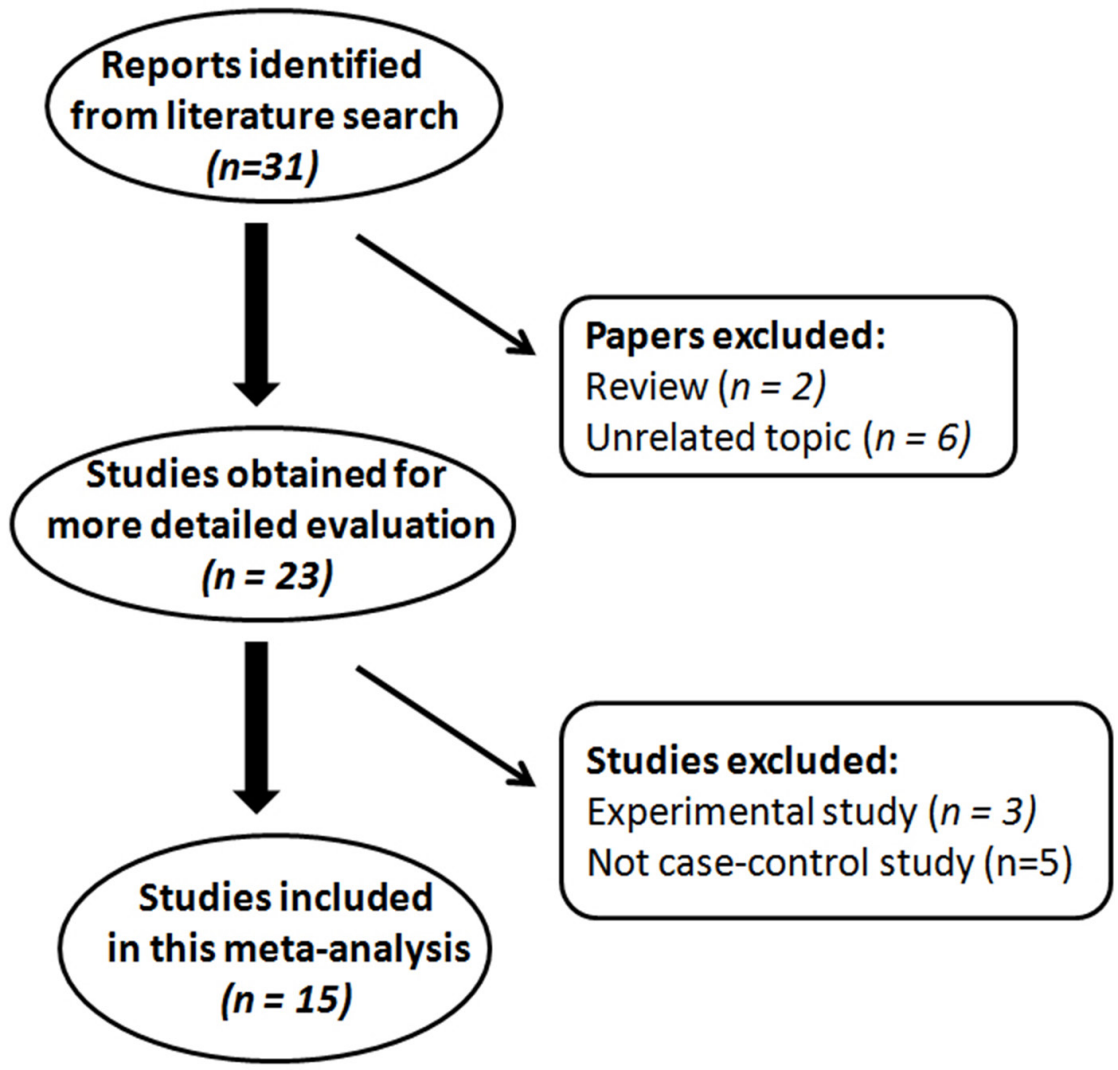
Flow diagram of literature search and study selection for meta-analysis

A flow diagram (Figure 1) shows the selection process of studies included in our analysis. The initial search strategy yielded 31 articles. Among them, two review articles were excluded because of the publication type and six articles were excluded because of unrelated topics. The full text of the remaining 23 studies was retrieved and reviewed. Eight articles were excluded after full-text review, five studies were not case-control studies and three articles were experimental studies. Finally, 15 articles were identified that met the inclusion criteria and included for the meta-analysis. The analyzed SNP was successfully genotyped and was within HWE (except the genotype in control subjects from the Takezaki's study) across all the included studies. All of the articles were case-control studies and all the cases were histopathologically confirmed as GC. The characteristics of these included articles are listed in Table 1 and Table 2. The NOS results showed that the score ranged from 7 to 8 with an average of 7.50, which indicated that the methodological quality of these selected articles was generally reliable.

**Table 1:** Characteristics of the fifteen studies included in this meta-analysis

Study	Country	Study population	Ethnicity	Study design		Genotyping method	Mean Age		NOS
				Case	Control		Case	Control	
Lu, et al. 2016	China	Chinese	Asian	CB	HB	SNaPshot	63.1 ± 10.7	63.3 ± 11.0	8
Hu, et al. 2015	China	Chinese	Asian	HB	HB	PCR-LDR	−	−	8
Engin, et al. 2011	Turkey	Turkish	Others	HB	HB	PCR-RFLP	60.4 ± 1.3	55.5 ±1.3	7
Liu, et al. 2011	China	Chinese	Asian	HB	CB	PCR-HMR	60.2 ± 10.4	59.3 ± 11.8	8
Canbay, et al. 2010	Turkey	Turkish	Others	CB	PB	PCR-RFLP	60.1 ± 20.9	52.8 ± 27.2	8
Sun, et al. 2010	China	Chinese	Asian	HB	PB	PCR-RFLP	59.6 ± 11.2	43.6 ± 10.3	7
Malik, et al. 2010	India	Indian	Asian	CB	PB	PCR-SSCP	55.9 ± 9.7	58.0 ±12.7	7
Palli, et al. 2010	Italy	Italian	Caucasian	CB	PB	Taqman	68.8 ± 9.9	55.5 ± 7.0	7
Farinati, et al. 2008	Italy	Italian	Caucasian	CB	PB	PCR-RFLP	68	46	8
Capella, et al. 2008	Spain	Spanish	Caucasian	CB	PB	Probe	50.5 ± 20.5	50.5 ± 20.5	8
Poplawskiet al. 2006	Poland	Polish	Caucasian	HB	PB	PCR-SSCP	62.4 ± 27.4	62.4 ± 27.4	8
Tsukino, et al. 2004	Japan	Japanese	Asian	HB	HB	PCR-SSCP	57.5 ± 9.5	57.1 ± 9.5	7
Takezaki, et al. 2002	China	Chinese	Asian	CB	PB	PCR- SSCP	65 ± 15	65 ± 14	7
Hanaoka, et al. 2001	Japan	Japanese Brazilian	Others	HB	HB	PCR-SSCP	65 ± 13	65 ± 12	7
Hanaoka, et al. 2001	Japan	non-Japanese Brazilian	Others	HB	HB	PCR-SSCP	59 ± 8	58 ± 8	8
Shinmura, et al. 1998	Japan	Japanese	Asian	HB	PB	PCR-SSCP	−	−	7

PB, population based; CB, clinic or institute based; HB, hospital based; NOS, Newcastle-Ottawa Scale; -Data unavailable.

**Table 2:** Genotype frequencies of hOGG1 rs1052133 among gastric cancer cases and controls in the included studies

Study	Sample size		Genotypes (Case)			Genotypes (Control)			Allele Frequency (G)		OR, 95%CI (Allellc Model)	*P*
	Case	Control	CC	CG	GG	CC	CG	GG	Case	Control		
Lu, et al. 2016	1279	1434	477	591	211	525	702	207	0.396	0.389	0.97 (0.87–1.08)	0.604
Hu, et al. 2015	436	372	154	210	72	128	176	68	0.406	0.419	1.06 (0.87–1.29)	0.586
Engin, et al. 2011	106	116	53	42	11	51	47	18	0.302	0.358	1.29 (0.87–1.92)	0.211
Liu, et al. 2011	618	913	114	302	202	144	447	322	0.571	0.594	1.10 (0.95–1.27)	0.199
Canbay, et al. 2010	40	247	24	13	3	171	69	7	0.238	0.168	0.65 (0.37–1.14)	0.131
Sun, et al. 2010	73	255	21	19	33	72	119	64	0.582	0.484	0.67 (0.46–0.98)	0.037
Malik, et al. 2010	108	195	50	51	7	94	89	12	0.301	0.290	0.95 (0.66–1.36)	0.772
Palli, et al. 2010	304	545	192	101	11	325	191	29	0.202	0.228	1.17 (0.92–1.49)	0.212
Farinati, et al. 2008	50	43	33	15	2	36	7	0	0.190	0.081	0.38 (0.15–0.95)	0.033
Capella, et al. 2008	438	1026	279	137	22	621	352	53	0.207	0.223	1.10 (0.91–1.34)	0.320
Poplawski, et al. 2006	28	33	22	6	0	18	15	0	0.107	0.227	2.45 (0.88–6.82)	0.079
Tsukino, 2004	142	271	32	75	35	74	141	56	0.511	0.467	0.84 (0.63–1.12)	0.232
Takezaki, 2002	101	198	20	61	20	30	120	48	0.500	0.545	1.20 (0.85–1.68)	0.292
Hanaoka, 2001	58	127	20	29	9	44	56	27	0.405	0.433	1.12 (0.72–1.75)	0.614
Hanaoka, 2001	208	205	133	67	8	123	74	8	0.200	0.220	1.13 (0.81–1.58)	0.480
Shinmura, 1998	35	42	9	16	10	15	20	7	0.514	0.405	0.64 (0.34–1.22)	0.174

MAF, minor allele frequency; OR, odds ratio; CI, confidence intervals; P, *p* value for *Z* test.

### Meta-analysis results

Fifteen studies provided results of the association of *hOGG1* rs1052133 with GC and a total of 10046 subjects (4024GC patients and 6022 controls) were tested for *hOGG1* rs1052133 in this meta analysis. Stratified analyses were conducted based on three groups, including Asian [11, 18, 24, 26, 27, 29–31], Caucasian [19, 21, 22, 25] and other ethnicities [20, 23, 28], and the heterogeneity of *hOGG1* rs1052133 in Asian, Caucasian, other and pooled populations were evaluated firstly. The results showed low heterogeneity for the included populations under all the genetic models by fixed-effect analysis, except for the Caucasian population under allelic genetic model (*P* = 0.052, *I^2^* = 61.2%), the Asian population under recessive model (*P* = 0.021, *I^2^* = 57.6%) and Caucasian population under dominant model (*P* = 0.054, *I^2^* = 60.7%), the random-effect analysis was thus adopted for them under each genetic model (Table 3).

**Table 3:** Pooled measure for the association of hOGG1 rs1052133 and gastric cancer different allelic models

Genetic model	Ethnicity	Pooled OR( 95% CI)	Heterogeneity		Test for overall effect		Begg test
			*P*	*I*^2^ (%)	*Z*	*P*	*P*▲
Allelic model ( C vs. G)	Asian	0.979 (0.879–1.091)	0.121	38.8	0.38	0.705	0.344
	CaucasianΔ	1.085 (0.789–1.492)	0.052	61.2	0.50	0.616	
	others	1.072 (0.841–1.366)	0.265	24.4	0.56	0.574	
	Overall	1.016 (0.924–1.117)	0.046	40.6	0.32	0.746	
Addictive model (CC vs. GG)	Asian	0.973 (0.839–1.130)	0.127	38.0	0.35	0.723	0.235
	Caucasian	1.171 (0.778–1.764)	0.351	4.5	0.76	0.450	
	others	1.210 (0.736–1.989)	0.261	25.0	0.75	0.452	
	Overall	1.010 (0.883–1.155)	0.186	24.2	0.14	0.888	
Recessive model (CC + GC vs. GG)	AsianΔ	0.939 (0.830–1.064)	0.021	57.6	1.01	0.313	0.322
	Caucasian	1.128 (0.752–1.692)	0.405	0.0	0.61	0.543	
	others	1.223 (0.765–1.956)	0.300	18.1	0.53	0.594	
	Overall	0.969 (0.864–1.087)	0.052	40.5	0.48	0.628	
Dominant model (CC vs. GG +GC)	Asian	1.039 (0.930–1.160)	0.692	0.0	0.68	0.498	0.260
	CaucasianΔ	1.136 (0.954–1.353)	0.054	60.7	1.43	0.152	
	others	1.079 (0.828–1.404)	0.470	0.0	0.56	0.575	
	Overall	1.067 (0.977–1.165)	0.408	4.0	1.45	0.147	

* OR, odds ratio; CI, confidence intervals; P, *p* value for *Z* test; *I*^2^ (%), the value to identify heterogeneity.

Δ Pooling model is random effect (Inverse Variance heterogeneity).

▲ Continuity corrected.

**Figure 2:**
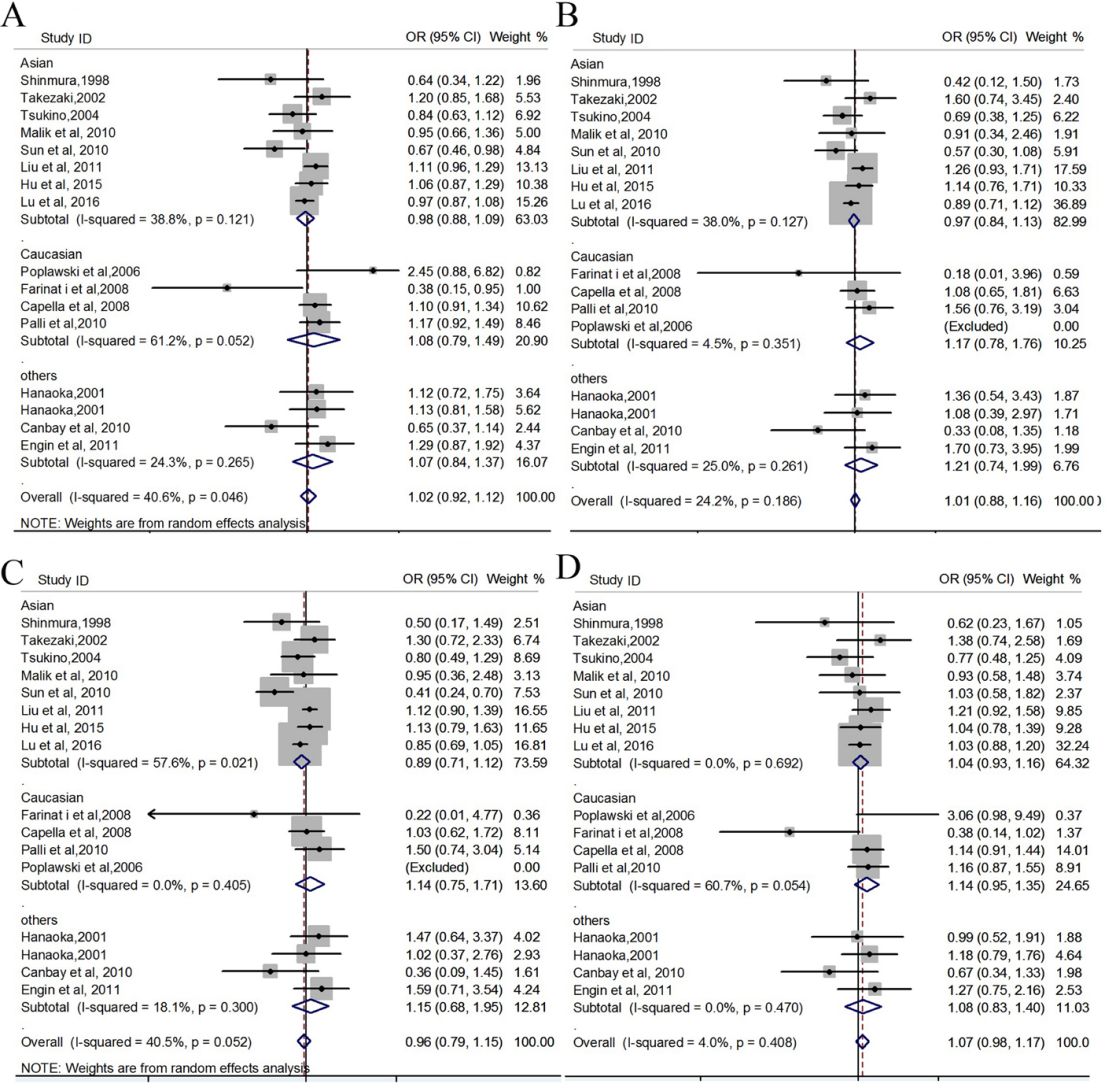
Forest plots for meta-analysis of *hOGG1* rs1052133 and the risk for GC (**A**) allelic model (C vs. G); (**B**) addictive genetic model (CC vs. GG); (**C**) recessive genetic model (CC + GC vs. GG); (**D**) dominant genetic model (CC vs. GG+GC).

Our meta-analysis showed that there was no significant association of *hOGG1* rs1052133 with GC in the overall pooled populations under all the genetic models (Allelic model: C vs. G, OR = 1.016, 95% CI 0.924 to 1.117, *p* = 0.746; *I^2^* = 40.6;Addictive model: CC vs. GG, OR = 1.010, 95% CI 0.883 to 1.155, *p* = 0.888; *I^2^* = 24.2; Recessive model: CC +GC vs. GG, OR = 0.969, 95% CI 0.864 to 1.087, *p* = 0.628; *I^2^* = 40.5; Dominant model: CC vs. GG+GC, OR = 1.067, 95% CI 0.977 to 1.165, *p* = 0.147; *I^2^* = 4; Table 3). Figure 2 shows the forest plot of estimates of odds ratios of the association of *hOGG1* rs1052133 with GC. When stratified analyses were conducted to explore further association of *hOGG1* rs1052133 with GC in different ethnicities under each genetic model, no significant association was detected between *hOGG1* rs1052133 and GC, either (Table 3 and Figure 2B–2D).

### Publication bias and sensitivity analysis

**Figure 3:**
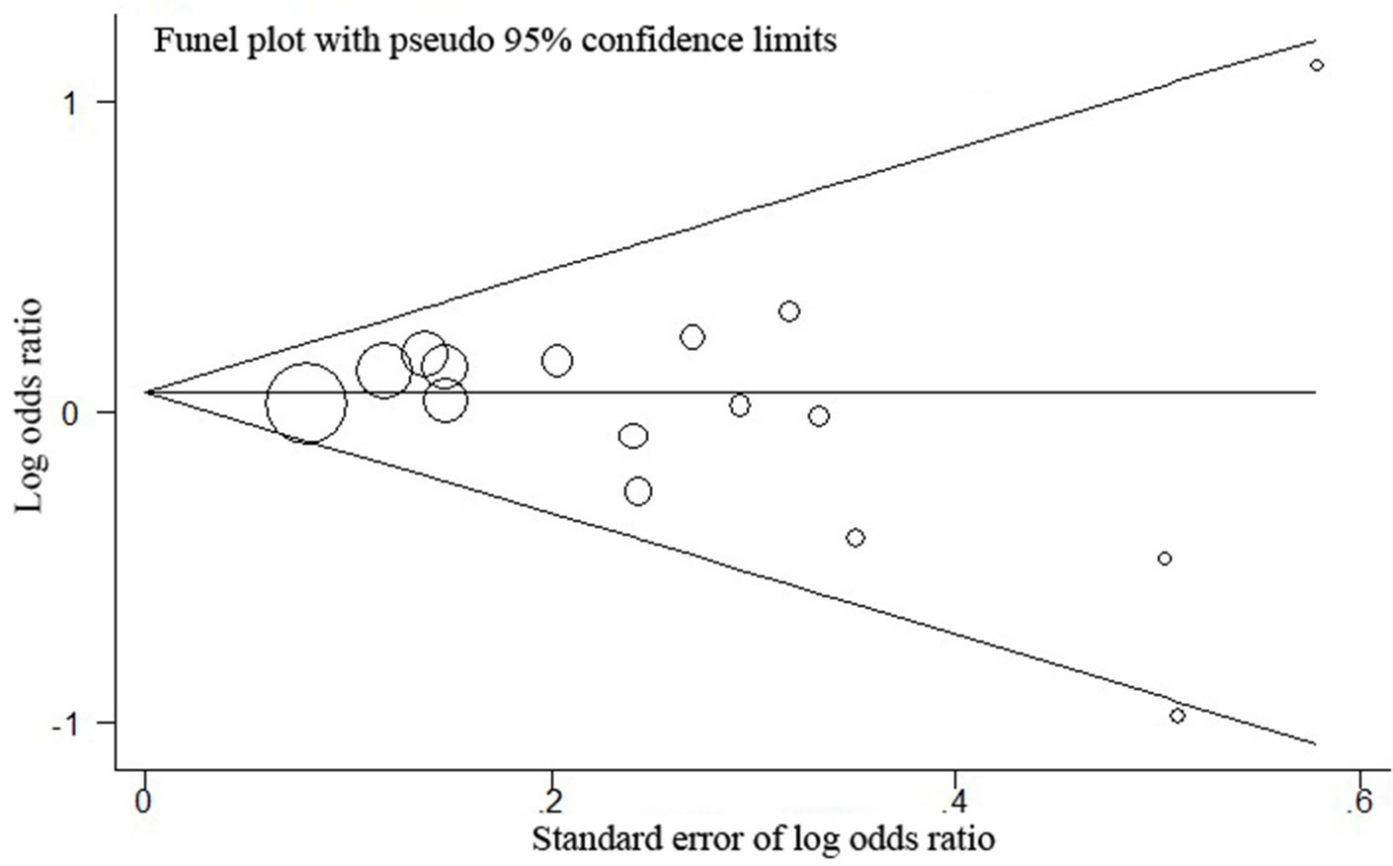
Funnel plot analysis for publication bias between *hOGG1* rs1052133 and GC risk P for publication bias of this funnel plot for dominant model (CCvs.GG+GC) with all 15 studies is 0.260.

Publication biases were assessed by the Egger's test quantitatively and the result for *hOGG1* rs1052133 based on the 15 included studies did not observe any obvious evidence of publication bias in the overall analyses under all genetic models (Table 3 and Figure 3). The effect of each study on the pooled OR was also assessed by sequential omission of individual studies. We did not find that the exclusion of any single study alter the significance of the final pooled OR.

## DISCUSSION

As a powerful statistical method, meta-analysis could provide a quantitative approach for pooling the variant results on the same topic to estimate and explain their diversity. This led us to conduct this meta-analysis of 15 published case-control studies, which may help us in distinguishing the truth from the false, and to explore a more robust estimate of the effect of *hOGG1* rs1052133 on GC. The *hOGG1* rs1052133 has been thought to constitute a candidate genetic risk factor for GC [11, 18, 19, 32]; however, several other studies have investigated the genetic effect of *hOGG1* rs1052133 on GC susceptibility with conflicting results [20–31]. In this study, our meta-analysis showed there was no association between *hOGG1* rs1052133 and GC in all genetic models, suggesting that *hOGG1* rs1052133 may not affect individual susceptibility to GC.

GC is considered to be a common, complex and multifactorial disease, and is estimated to have a significant heritable component. GC ranks as the third leading cause of cancer mortality worldwide and confers a 5-year survival of 20% [33]. Identifying causative genes has helped to understand the disease process and may aid in prevention. About 1%–3% of GC could be attributed to inherited cancer predisposition syndromes. More advances have been made in human GC genetics, but there is still to be known [8]. To date, Germline E-cadherin/CDH1 mutations have been identified in families with an autosomal dominant inherited predisposition to diffuse GC [34]. *hOGG1* has been reported to be related to GC risk in several studies [11, 18, 19, 32]. It is an important glycosylase enzyme that plays a critical role in the repair of DNA oxidative damage [14, 15]. The dysfunction of hOGG1 might lead to deficiency of DNA repair and result in cell canceration. hOGG1 variant has been shown to exhibit lower DNA repair activity and greater sensitivity to oxidation than wildtype hOGG1 protein [16].

The association between *hOGG1* rs1052133 and GC in previous studies were inconsistent. *hOGG1* rs1052133 was reported to be significantly associated with GC in Japanese and Chinese patients with GC [11, 18, 19, 32]. However, no evidence of association between *hOGG1* rs1052133 and GC was detected in Turkish, Spanish, Italian, Polish and Indian studies [21–25, 28], meanwhile, the association of *hOGG1* rs1052133 with GC was not replicated in other Japanese and Chinese populations [20, 26, 27]. Recently, the association between *hOGG1* rs1052133 with GC risks was also accessed in Lu *et al*’ study with 1,275 GC patients and 1,436 controls [29] and in Hu *et al*'s study with 2745 GC patients and 4588 controls [30], respectively. Both the two studies demonstrated that the significant association was not present between *hOGG1* rs1052133 and GC. In order to provide powerful statistical analysis, we conduct this study with more samples (a total of 4024GC patients and 6022 controls) to evaluate the association between *hOGG1* rs1052133 and GC. Our results did not show any significant association of *hOGG1* rs1052133 with GC, even across different ethnic populations. Therefore, our analyses failed to conclude whether *hOGG1* rs1052133 is really a GC-associated SNP and more replication data is needed to validate this association. Of course, other factors, such as environment and different lifestyle, might play roles in these differences as well. Further analysis should be performed in more large-scale cohorts or case-control studies to explore the association of *hOGG1* rs1052133 with GC. Future studies on gene-environment interaction should also be considered.

This systematic review and meta-analysis has sufficient power to evaluate and review all the published genetics studies in *hOGG1* rs1052133, however, some potential limitations of our study on the understanding of *hOGG1* in GC genetics should be considered. (1) This meta-analysis was mainly based on the studies with 2950 GC patients and 3817 controls in Japanese and Chinese populations [11, 18, 20, 26, 27, 29–31]. Besides, only 1074 GC patients and 2205 controls in Turkish, Indian, Italian, Spanish and Polish populations were included in the present study. It may restrict our conclusions which indicate the need for larger sample sizes in other ethnic populations. (2) There was a high heterogeneity detected in the Caucasian population under allelic genetic model, in the Asian population under addictive model and in the Caucasian population under dominant model. It is possible that different populations with different clinical patient characteristics were included. (3) GC is a multifactorial disease that results from complex interactions between various genetic factors and other factors. Therefore, our results may be influenced by confronting factors, such as age, gender, environment, and lifestyle. If the investigation of gene-environment interactions in different ethnic subgroups could be carried out, we might get more conclusive claims about the association of *hOGG1* rs1052133 with GC.

In conclusion, this meta-analysis showed that *hOGG1* rs1052133 is not associated with GC under all the genetic models, suggesting that it may not affect individual susceptibility to GC. Further investigations with larger sample sizes and more ethnic groups are required to validate the association and confirm the roles of *hOGG1* in GC.

## MATERIALS AND METHODS

### Searching strategy

A systematic literature search using the databases PubMed, Embase, Web of Science, and the Chinese Biomedical Database was conducted, to identify all published studies on the association of *hOGG1* polymorphisms with GC from their starting date to December 15, 2016. Following keywords were used: ‘‘cancer,’’ ‘‘gastric cancer,’’ ‘‘human 8-oxoguanine glycosylase,’’ ‘‘hOGG1,’’ ‘‘rs1052133,’’ ‘‘polymorphism(s),’’ ‘‘variant(s),’’ and ‘‘mutation(s).’’ The internet was searched using the Google search engine. Reference lists of the retrieved articles and reviews were manually checked for additional articles, to identify studies not yet included in the electronic searches.

### Inclusion and exclusion criteria

The eligible articles were considered if they (1) evaluated associations between *hOGG1* rs1052133 and GC; (2) used a case–control design to compare GC cases and normal controls in defined populations; (3) gave an OR with 95% CI or other available data from which they could be estimated; and (4) were original research articles, not reviews or comments. Excluded were abstracts from conferences, full texts without raw data available for retrieval, republished data, duplicate studies and reviews.

### Data extraction

Two observers (DDZ and XNG) independently abstracted data from all eligible publications onto paper data collection forms. Two reviewers (JLH and GQZ) were blinded to the details (title, author, and academic address) of these studies during assessment. Disagreements were resolved by discussion until a consensus was achieved. Otherwise, a third investigator was consulted to resolve the dispute. The following items were collected from each study: first author's surname, year of publication, statistical data, ethnicity of subjects, whether Hardy-Weinberg equilibrium (HWE) was examined in controls, genotyping method, total numbers of cases and controls, as well as total numbers of cases and controls for each *hOGG1*genotypes, respectively.

### Quality assessment

The Newcastle-Ottawa Scale (NOS) was used to assess the quality of the included studies by two investigators independently. Through the rating system, the NOS was conducted to judge the study quality based on three aspects, which were selection, comparability, and exposure situation in case-control studies. Rating scores range from 0 (worst) to 9 (best). Studies with a score of 7 or greater were thought to have an adequate or good quality.

### Statistical analysis

A pooled OR with its corresponding 95% CI was used as a measure of the association of *hOGG1* rs1052133 with GC. For genotypic comparison, allelic, addictive, dominant and recessive models were applied in the investigation of the disease association. We conducted stratified analyses by ethnicity, including Asian populations (Japanese, Chinese and Indian), Caucasian populations (Polish, Italian and Spanish), other populations (Turkish, Japanese Brazilian and non-Japanese Brazilian) and pooled populations. Sensitivity analysis was carried out by excluding one study at a time to explore whether the results were influenced by a specific study. Heterogeneity (between-study inconsistency) was investigated and measured using *I^2^* statistic. A *p* value of *I^2^* < 50% indicated an absence of heterogeneity among studies, the fixed-effect model (Mantel-Haenszel method) was thus used to calculate the pooled ORs. In contrast, if the *p* value for heterogeneity was *I^2^* ≥ 50%, indicating a high degree of heterogeneity between studies, then the random-effect model (DerSimonian-Laird method) was used to evaluate the summary ORs. Begg liner regression test was used to assess the potential publication bias, where a value of *p* < 0.05 was considered statistically significant. The Hardy-Weinberg equilibrium (HWE) for each SNP was tested by the χ^2^ test. All statistical analyses were performed by the Review Manager software (RevMan, version 5.2; The Nordic Cochrane Centre, The Cochrane Collaboration, Copenhagen;2012), and the STATA software (version 12.0, STATA Corp., College Station, TX, USA), as well as the Hardy Weinberg package (version 1.3) in R language (version 2.15.0,http://cran.r-project.org/). Two-sided *P* values < 0.05 were considered statistically significant.
